# Risk Factors for Human Lice and Bartonellosis among the Homeless, San Francisco, California, USA 

**DOI:** 10.3201/eid2010.131655

**Published:** 2014-10

**Authors:** Denise L. Bonilla, Charsey Cole-Porse, Anne Kjemtrup, Lynn Osikowicz, Michael Kosoy

**Affiliations:** California Department of Public Health, Richmond, California, USA (D.L. Bonilla);; California Department of Public Health, Sacramento, California (C. Cole-Porse, A. Kjemtrup);; Centers for Disease Control and Prevention, Fort Collins, Colorado, USA (L. Osikowicz, M. Kosoy)

**Keywords:** Bartonella quintana, homeless, body lice, head lice, trench fever, vector ecology, San Francisco, epidemiology

## Abstract

Homeless persons in San Francisco, California, USA, have been shown to have head and body lice infestations and *Bartonella quintana* infections. We surveyed a self-selected population of homeless persons in San Francisco to assess infestations of head and body lice, risks of having body lice, and presence of *B. quintana* in lice. A total of 203 persons who reported itching were surveyed during 2008–2010 and 2012: 60 (30%) had body lice, 10 (4.9%) had head lice, and 6 (3.0%) had both. *B. quintana* was detected in 10 (15.9%) of 63 body lice pools and in 6 (37.5%) of 16 head lice pools. Variables significantly associated (p<0.05) with having body lice in this homeless population included male sex, African–American ethnicity, and sleeping outdoors. Our study findings suggest that specific segments of the homeless population would benefit from information on preventing body lice infestations and louseborne diseases.

## Abstract 2

Results suggest that body lice disproportionately affect certain demographic groups and those who sleep outdoors.

The human body louse (*Pediculus humanus humanus*) has played a key historical role in the transmission of diseases such as trench fever, epidemic typhus, and louseborne relapsing fever (*1*,*2*). Because of the emergence of pesticide-resistant head lice (*P. humanus capitis*) (*3*), lice continue to be part of the human landscape. Head lice are not implicated in the transmission of disease-causing agents, but they do have social and economic effects because of the school days missed by children with infestations (*3*). Like head lice, pubic lice (*Pthirus pubis*) are not known to transmit pathogens (*4*). Although diseases are not transmitted by all lice species, heavy louse infestations can cause pruritus, secondary infections from scratching, and anemia (*5*,*6*).

A combination of conditions, including poor clothing hygiene, lack of resources, and cold weather, put certain human populations, such as homeless persons, more at risk of harboring lice and for louseborne diseases (*2*). Research suggests that environments frequented by homeless persons (e.g., homeless shelters, where there is close body-to-body contact and where clothing hygiene is lacking) promote louse transmission (*2*,*5*). The number of homeless persons is not trivial. A single night count by the Annual Homeless Assessment Report (AHAR) estimated 649,917 homeless persons in the United States in 2010; of that total, 5,823 (0.9%) were in San Francisco (*7*). Given the large number of homeless persons in the United States, human lice of all species and the diseases they may carry are a health concern for this population.

Of concern is the potential for body lice to transmit *Bartonella quintana,* the bacterium that causes trench fever, the most common louseborne disease in some urban homeless persons (*8*). Trench fever is characterized by severe frontal headache, dizziness, conjunctival congestion, shin pain, and lymphadenopathy, accompanied by a relapsing fever lasting 4–8 days at a time. Life-threatening complications, such as endocarditis and bacillary angiomatosis, can occur (*1*,*9*). The only known vector of *B. quintana* is the body louse, although recent studies suggest head lice may also vector disease agents (*10*–*12*). *B. quintana* proliferate in body lice 4 days after ingestion and are continuously excreted in feces for >3 weeks (*13*). *B. quintana* can be transmitted to a human host when an infected louse feeds on an uninfected human and excretes *B. quintana-*infected feces onto their skin (*1*). The bacteria are rubbed into open mucous membranes or scratched into the skin through the bite wound (*5*), resulting in *B. quintana* infection 15–25 days later (*1*). Although *B. quintana* are principally infective to humans, macaque monkeys and the lice that infest them can maintain and circulate a strain of *B. quintana* (*14*).

In a previous study of head and body lice from homeless persons in San Francisco, we determined that 33.3% of body lice samples and 25% of head lice samples, which were pooled by host, were positive for *B. quintana* (*12*). To better characterize body and head lice transmission and to gauge the risk for *B. quintana* infection, we evaluated San Francisco homeless persons to determine presence of head and body lice, assess habits that may increase the risk of acquiring lice, and collect lice to test for *B. quintana* infection.

## Methods

### Survey and Lice Collection

Methods for this study were reviewed and approved by the State of California Committee for the Protection of Human Subjects (protocol no. E-108-10-01). The California Department of Public Health, Vector-Borne Disease Section, participated in San Francisco’s Project Homeless Connect (SFPHC), under the auspices of SFPHC medical services on 9 separate dates in 2008–2010 and 2012. In addition to providing the lice-related services described below, we administered a 15-question survey regarding lice transmission to all consenting homeless adults who inquired about services. Questions pertained to where the person slept, frequency of sharing/swapping used clothing, and prior exposure to lice. No personal identifiers were recorded. Persons came directly to the Vector-Borne Disease Section booth or by way of medical triage when they reported itching or “bug” problems. Persons requesting lice-related services received services regardless of whether they agreed to participate in the survey.

Lice-related services included examination of hair, body, and clothing for ectoparasites. Lice in the clothing or on the body below the neck were considered a positive indication of body lice infestation. Persons with body lice infestations were re-clothed to remove the source of lice. Those with infestations of more than a few lice were provided a shower and clean clothes. Infested persons were given verbal counseling on the control of body lice and a body lice fact sheet, and they were then accompanied to a licensed medical practitioner to complete their medical review.

Finding live lice on the head or hair, with the presence of nits, was considered a positive indication for a head lice infestation. If a person had live head lice or >20 nits that were within a quarter inch of the scalp, they were given a kit with 1% permethrin lotion and a nit-remover comb, and were counseled on how to properly use the kit. Persons with severe head lice infestations or with matted hair were offered free haircuts.

The body and head lice that were collected from all persons were processed as previously described (*12*). In brief, <20 lice of each species were collected from each person and placed, by identifying number and species, into tubes with 70% ethanol. In the laboratory, both lice species were counted and placed into clean tubes with 70% ethanol; each person’s pool contained 1–20 lice. For each person, only 1 pool of each louse species was tested. The lice pools were sent to the *Bartonella* Laboratory at the Centers for Disease Control and Prevention, Fort Collins, Colorado, USA, for DNA extraction, PCR testing, and sequencing.

### Detection of *Bartonella* by PCR

Sixteen pools of head lice and 63 pools of body lice were homogenized by using a Mixer Mill MM 200 (Retsch, Newtown, PA, USA). The homogenate was prepared for DNA extraction by using a QIAamp DNA Mini Kit (QIAGEN, Valencia, CA, USA), following the manufacturer’s tissue protocol.

PCR was performed in a TaKaRa PCR Thermal Cycler Dice (Takara Bio Inc., Shiga, Japan) targeting the citrate synthase gene (*gltA*) and the 16S-23S rRNA intergenic transcribed spacer region (ITS), using previously described primers (*15*,*16*). The PCR cycle conditions for *gltA* were 2 min at 95°C, followed by 38 cycles of 30 s at 95°C, 30 s at 48°C, 2 min at 72°C, and an extension for 7 min at 72°C. The conditions for ITS were 3 min at 95°C, followed by 55 cycles of 30 s at 95°C, 30 s at 66°C, 30 s at 72°C, and an extension for 7 min at 72°C. *B. doshiae* DNA was used as a positive control and nuclease-free water was used as a negative control.

The PCR amplicons were purified by using a QIAquick PCR Purification Kit (QIAGEN) and were sequenced on a 3130 Genetic Analyzer (Applied Biosystems, Foster City, CA). Sequence data were analyzed by using Lasergene 9 software (DNAstar, Madison, WI, USA) and aligned with *Bartonella* type strains from GenBank.

### Data Analysis

All statistical analyses were conducted by using R statistical software (RStudio version 0.97.336; http://www.rstudio.com/); p values <0.05 were considered significant. The analysis included data from all persons who had a body lice examination and who agreed to participate in the survey. Persons were classified as having body lice or not having body lice, on the basis of the outcome of the examination. The explanatory variables analyzed in relation to body lice presence follow: reason for requesting services (bugs, itch, lice, or free shirt), sex, age, race/ethnicity, amount of time homeless (<1 year, 1–5 years, >5 years), where they slept (specific San Francisco district), whether they slept indoors or outdoors, frequency of sleeping in close proximity to others, frequency of clothing exchanged, and previous exposure to lice. We conducted univariate analysis, using χ^2^, for each explanatory variable to test for association with the outcome of having body lice. Explanatory variables that were determined to have a significant association (p<0.05) with a person having body lice were included in a generalized linear model to further analyze the predictive value of the variables for the outcome of having body lice. Having body lice or not was coded as a binary variable, and a logistic regression was run by using a backward process. The initial model contained all the significant variables from the univariate analysis. Variables were removed from the model, and their change effect was observed. The change in Akaike information criterion was also used as an indicator for the model’s predictive value.

Survey participants co-infested with head and body lice (n = 6) were included in the body lice analysis. Data from the few persons with head lice only (n = 10) were analyzed separately, and a descriptive summary is provided.

## Results

### Survey

In all, 203 persons received an examination and completed a survey. Of these 203 persons, most (145, 71%) were men. The median age was 46 years (range 19–68). The racial/ethnic make-up was 91 (45%) white, 51 (25%) African American, 28 (14%) Hispanic, 24 (12%) Native American, and 9 (4%) Asian–Pacific Islander.

Survey participants reported spending most of their time in 19 different San Francisco districts or neighborhoods that are spread throughout the city; a 50-square-block area in north-central San Francisco (the Tenderloin district) was reported most frequently (76/203 reports, 37%). Of the 203 persons, 134 (66%) reported regularly sleeping indoors, including in shelters, single resident occupancies, and motels or other establishments; 65 (32%) reported sleeping outdoors in public locations; and 4 (3%) did not provide responses. Survey participants reported being homeless for <1 year (93, 46%), 1–5 years (53, 26%), or >5 years (36, 18%). Twelve (6%) of the 203 survey participants reported not being homeless, and 9 (4%) did not respond to the question. Surveys from these 21 persons were included in the analysis because, by participating in SFPHC, they were considered part of the homeless community.

Of the 203 survey participants, 132 (65%) reported never exchanging clothes with others, 35 (17%) reported doing so once a month or “sometimes,” 23 (11%) reported doing so >4 times a month or “frequently,” 9 (5%) reported doing so 2–3 times a month or “often,” and 4 (2%) did not respond. Of the 150 persons who responded to a question about sleeping distance from another person, 75 (37%) reported never sleeping within an arm’s length of another person, and 75 (37%) reported always doing so. Slightly over half of the survey participants (107, 53%) reported previously having lice.

### Head Lice

Ten of the study participants had head lice only, and 6 had head and body lice. Of the 10 with head lice only, the mean age was 46 years (range 22–58 years), 4 (40%) were male, 7 (70%) reported race/ethnicity as white and 3 (30%) as Native American, and all 10 reported having had lice before. Of the 10 persons with head lice only, 3 (30%) reported sleeping in the Tenderloin district, and 6 (60%) reported sleeping indoors, including at single resident occupancies and hotels. None of the 10 reported sleeping in a shelter. Three (30%) of the 10 persons reported never sleeping in close proximity to others, and 3 (30%) reported always sleeping in close proximity to others. Most of those with head lice only (7/10, 70%) reported never exchanging clothing.

### Body Lice

Of the 203 persons who received a body lice examination, 60 (30%) were found to have body lice, including 6 who were co-infested with head lice. Table 1 shows a list of explanatory variables that were compared between persons with and those without body lice to determine an association with body lice infestation. Significant differences were found for sex, race/ethnicity, sleeping location, and the reason for the examination. Male sex, African–American ethnicity, sleeping outside, and lice being the reason for requesting services were more frequent among persons with body lice than among those without body lice (Table 1). In the final generalized linear model with the highest predictive value, the following were significantly associated with having body lice: male sex, African American ethnicity, sleeping outdoors, and lice as the reason for requesting services (Table 2).

**Table 1:** Association of body louse infestation and explanatory variables in a homeless population surveyed during 2008–2010 and 2012, San Francisco, California, USA

Variable	No. (%) with body lice (n = 60)	No. (%) without body lice (n = 143)	p value*
Sex			0.00†
Male	55 (91.7)	90 (62.9)	
Female	5 (8.3)	52 (36.4)	
No response	0	1 (0.7)	
Race/ethnicity			0.05†
White	27 (45.0)	64 (44.8)	
African American	21 (35.0)	30 (21.0)	
Hispanic	6 (10.0)	22 (15.4)	
Native American	4 (6.7)	20 (14.0)	
Asian	2 (3.3)	7 (4.9)	
Age group			0.19
19–28 y	1 (1.7)	11 (7.7)	
29–38 y	7 (11.7)	20 (14.0)	
39–48 y	27 (45.0)	43 (30.1)	
49–58 y	19 (31.7)	54 (37.8)	
59–68 y	5 (8.3)	15 (10.5)	
No response	1 (1.7)	0	
Sleeping			0.01†
Inside	30 (50.0)	104 (72.7)	
Outside	30 (50.0)	38 (26.6)	
No response	0	1 (0.7)	
Exchange clothing			0.76
Frequently	6 (10.0)	17 (11.9)	
Often	3 (5.0)	6 (4.2)	
Sometimes	8 (13.3)	27 (18.9)	
Never	41 (68.3)	91 (63.6)	
No response	2 (3.3)	2 (1.4)	
Time homeless			0.97
<1 y	29 (48.3)	64 (44.8)	
1–5 y	15 (25.0)	38 (26.6)	
>5 y	11 (18.3)	25 (17.5)	
Not homeless	3 (5.0)	9 (6.3)	
No response	2 (3.3)	7 (4.9)	
Reason for examination			0.02†
Bugs	5 (8.3)	28 (19.6)	
Itch	32 (53.3)	50 (35.0)	
Lice	18 (30.0)	37 (25.9)	
Services (free shirt)	5 (8.3)	28 (19.6)	

*p value of χ^2^ test evaluating the distribution of variables between those with and without body lice.  †Indicates the distribution of the variable is significantly (p<0.05) different between groups.

**Table 2:** Results of logistic regression for the association of body louse infestation with certain explanatory variables in a homeless population surveyed during 2008–2010 and 2012, San Francisco, California, USA

Coefficient	Estimated values	SE	p value	Odds ratio	95% CI
Intercept	−3.82	0.73	<0.01	Reference*	–
Sleeping outdoors	0.98	0.37	0.01	2.66	1.29–5.59
African American†	1.03	0.40	0.01	2.81	1.30–6.23
Male	1.73	0.52	0.00	5.64	2.19–17.57
Reason for requesting services, lice‡	1.22	0.60	0.04	3.39	1.09–1.21

*Reference group is female, who sleep indoors, of ethnic self-reported background other than African American, and who requested services for reasons other than body lice. †All other races/ethnicities combined (white, Hispanic, native American, and Asian) was used as the reference group for this variable on the basis of the results of the univariate analysis ‡All other reasons for requesting services (bugs, itch, and services, such as t-shirts) was used as the reference group for this variable on the basis of the results of the univariate analysis

### Lice Testing

Sixty pooled body lice samples, from 60 persons, were tested by PCR. In addition, 3 pooled samples from 3 persons without surveys were also tested. Of the 63 body lice pool samples, 10 (16%) were positive for *B. quintana*. Of the 7 surveyed persons with *B.* quintana–positive body lice, 6 (86%) were male, 5 (71%) were white, 1 (14%) was African American, and 1 (14%) was Native American. Of those 7 persons, 5 (71%) reported sleeping outdoors and 2 (29%) reported sleeping indoors.

Of 16 head lice pools, 6 (38%) were positive for *B. quintana*. All head lice samples came from persons who completed questionnaires. Of the 6 persons with positive samples, 4 (67%) were male, 5 (83%) were white, and 1 (17%) was Native American. Of those 6 persons, 4 (67%) reported sleeping outdoors and 2 (33%) reported sleeping indoors.

None of the persons with co-infestations (n = 6) had head and body lice positive for *B. quintana*. However, the head lice pool for 1 co-infested person was positive, and the body lice pool for another co-infested person was positive for *B. quintana*.

*The B. quintana* *gltA* sequences that we obtained were not distinguishable from a sequence previously submitted to GenBank (accession no. U28073). The ITS sequences that we obtained also were not distinguishable from a sequence previously submitted to GenBank (accession no. AF368391).

## Discussion

By combining body lice examinations with a behavioral questionnaire in this study, we documented demographic and behavioral factors associated with an increased risk of acquiring body lice in this self-selected population of homeless persons. We found that 30% of persons in our study, most of whom sought services for possible lice infestation, had body lice. In other homeless populations, a body lice infestation prevalence of 7%–22% has been reported (*8*,*17*). However, the 16% pooled prevalence of *B. quintana* in the recovered lice in our study is similar to that in a previous study of a similarly selected group from the homeless population in San Francisco (*12*). This prevalence, along with the knowledge that body lice can transmit *B. quintana* (*8*), is of concern and suggests that bartonellosis (trench fever) is a risk among these homeless persons.

The results from our questionnaire suggest additional avenues of investigation to improve body lice prevention efforts. Male sex, African–American ethnicity, sleeping outdoors, and lice as reason for requesting services were significantly (p<0.05) associated with having body lice in our study population of homeless persons (Table 2). Reasons such as inadequate access to resources or differences in risk behavior may explain the demographic factors that were significant (*18*). In a January 2012 point-in-time estimate, it was determined that 57.2% of the San Francisco homeless population was unsheltered, compared with 38% across the United States (*19*). We showed that persons sleeping outdoors had an increased risk for body lice. Several of the persons in this study who slept outdoors noted having lice-infested sleeping bags. Studies conducted in France found that shared bedding in shelters is a key factor in lice transmission (*9*,*17*). Thus, the potential for louse transmission through shared outdoor bedding (i.e., sleeping bags) warrants further investigation.

The length of time being homeless was not associated with body lice infestation in our study. This finding is similar to that in a previous study in which the amount of time a person had been homeless was not significantly associated with louse infestation among 126 homeless persons from emergency departments and shelters in Marseille, France (*20*). In the same study, Foucault et al. (*20*) found that the presence of body lice was associated with *B. quintana* bacteremia. Although we could not assess *B. quintana* infection in the persons in our study, we did document that the body lice from 10 (16%) of 63 body lice–infested persons were positive for *B. quintana*, suggesting an infection risk for these persons. Both studies underscore that lice infestations, and potential *B. quintana* infection, can occur at any time during a period of homelessness.

Persons who are unaware of a personal lice infestation may contribute to body lice transmission cycles in ways that have not been examined. In this study, there was no significant difference in lice infestation between those who reported sharing/swapping clothes and those who did not report such behavior. However, participants’ answers to this question could be biased by a reluctance to admit wearing unwashed, used clothing. Persons came to our booth when they believed they had a problem, so we were not able to estimate the prevalence of lice infestation in the homeless population overall.

From the time *B. quintana* DNA was first detected in head lice, these lice have been of interest in studies of bartonellosis and of *B. quintana* transmission, but the epidemiologic role of head lice is still unclear (*10*, *12*,*21*). The percentage of *B. quintana–*positive head lice pools (37.5%) in the current study was slightly higher than the percentage we reported in 2007–2009 (22.2%) (*12*), but the difference was not statistically significant. *B. quintana* has been detected from head lice collected from street beggars in Ethiopia (9.2%) and from street children in Nepal (9.5%), although some of the reported head lice infections in those studies may have been due to co-infestations of body and head lice (*10*,*21*). The presence of *B. quintana* in head lice and its absence in body lice (and vice-versa) from the same person may be due to the phenomenon of niche-sharing. In a recent genetic study of head and body lice collected from homeless persons (*22*), it was hypothesized that in cases of massive infestations of body and head lice, the lice will wander into the ecologic niches of the opposing lice, possibly evolving to colonize the new niche (*22*,*23*). Although we tried to collect lice that were clearly regionalized to the head (above the neck and with hair nits) or body (below the neck with clothing nits), the 2 types of lice could have migrated between regions and thus have been misclassified as body lice and vice versa. In our study, 6 persons had co-infestations of head and body lice, but none of these persons had both body and head lice positive for *B. quintana*. Further studies on body and head lice should genetically characterize lice species.

The nature of this study presented several limitations. First, our analysis and results are only generalizable to persons seeking lice-related services and may or may not be applicable to other members of the homeless community. Second, in several instances, we performed examinations of and collected lice from persons who did not participate in the survey but who had lice positive for *B. quintana*. Information for these persons was not available for the analysis conducted in this study, and such data would have further enhanced our understanding of the risk for *B. quintana* infection and the risk of acquiring lice. Third, although B. quintana–infected head lice were identified (37.5%), a small sample size precluded conclusions about the risk of acquiring head lice in this group or the role head lice may play in bartonellosis epidemiology. Last, the study protocol did not include the collection and testing of serum samples from survey participants, so we could not assess the risk that the presence of body lice, let alone the presence of infected body lice, specifically poses to human infection with *B. quintana*. Nonetheless, results from this study can influence public outreach messaging that sleeping outdoors is a risk behavior for the acquisition of body lice among the homeless. Although we did not identify a specific behavior associated with outdoor sleeping that could transmit lice, shared bedding (i.e., sleeping bags) may be a factor in the transmission of body lice for homeless persons who sleep outdoors.

The results from our study showed a relatively high prevalence of body lice (30%) in this group of homeless persons who self-selected for lice-related services. In addition, 16% of the recovered lice were infected with *B. quintana*, suggesting that this population is at risk for bartonellosis. The presence of body lice was positively associated with male sex, African–American ethnicity, and sleeping outdoors. Our findings suggest that focusing prevention information, such as promoting use of clean sleeping bags or explaining how to clean bedding, to those who sleep outside may be of additional benefit for decreasing lice infestations, and this possibility warrants further investigation.
